# Neighborhood green space visits and coronary heart disease: Evidence from mobility data across nine U.S. metropolitan cities

**DOI:** 10.1016/j.ajpc.2026.101666

**Published:** 2026-05-14

**Authors:** Sarayu Chandra Mouli, Tong Zhang, Jad Ardakani, Zhuo Chen, Sanjay Rajagoaplan, Jay Maddock, Khurram Nasir, Weichuan Dong, Sadeer Al-Kindi

**Affiliations:** a Center for Health & Nature, Houston, TX, United States; b School of Medicine, Case Western Reserve University, Cleveland, OH, United States; c Department of Cardiology, Houston Methodist, Houston, TX, United States

**Keywords:** Nature, Greenspace visits, Mobility, Cardiovascular health, Spatial heterogeneity

## Abstract

**Background:**

Coronary heart disease (CHD) is the leading cause of mortality and morbidity globally and it is shaped by genetics, behaviors and the environment. While emerging evidence suggests that greenspaces can reduce cardiovascular morbidity, most studies rely on static vegetation indices (e.g., NDVI) that do not account for greenspace engagement. This study bridges that gap to examine how greenspace engagement relates to CHD, accounting for metabolic and social factors.

**Methods:**

Green space visits per person was derived from Advan Research data (2021) at the census tract-level across the nine largest U.S. metropolitan areas (MSAs) and linked with CHD prevalence and risk factors from CDC PLACES (2023). Ordinary least-squares (OLS) models assessed metro-specific associations between greenspace visits (10 visits) and CHD, adjusting for demographics, social vulnerability, Normalized Differential Vegetation Index (NDVI), and metabolic factors. Geographically weighted regression (GWR) assessed within-metro spatial variation.

**Results:**

Across the nine MSAs, higher greenspace visitation was associated with lower CHD prevalence. Each additional 10 greenspace visits per person corresponded to a 1.6 % lower CHD prevalence in Houston, 1.6 % in Atlanta, 1.5 % in Chicago, 1.5 % in Dallas, and 1.1 % in Phoenix (all *p* < 0.01). Los Angeles (+0.4 %) and Philadelphia (+0.9 %) demonstrated small positive relationship with CHD. GWR revealed variations in these associations within each Metro area, with suburban areas showing strongest negative relationship between green space visits and CHD prevalence.

**Conclusions:**

Greenspace engagement was broadly associated with lower CHD prevalence, with notable variations among cities and across neighborhoods within each city. Strong inverse associations were noted in suburban areas and neighborhoods with elevated metabolic risk, whereas urban cores showed weaker patterns.

**Figure:**
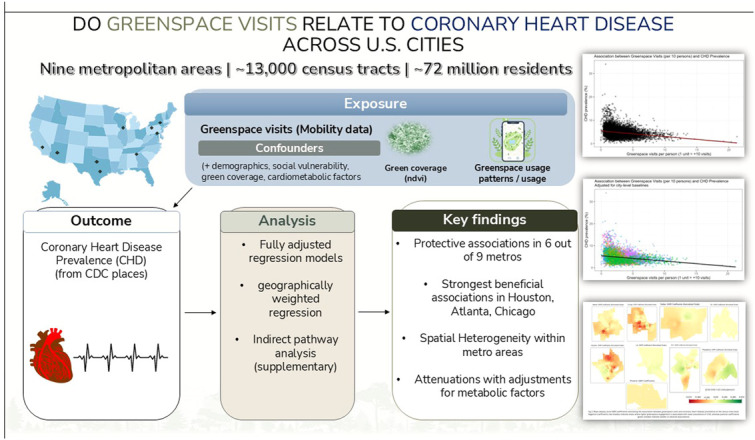
Central illustration.

## 1 Introduction

Coronary heart disease (CHD) remains the leading cause of mortality in the United States and worldwide, accounting for nearly one in every five deaths [1]. Despite major advances in prevention and treatment, CHD continues to reflect deep social, behavioral, and environmental inequities [2,3]. Individual risk factors such as smoking, hypertension, diabetes, and obesity are well established, yet these determinants are shaped by the broader environments in which people live, work, and engage in daily activity [[4], [5], [6], [7]]. Recognizing this, a growing body of evidence increasingly links CHD outcomes to nature and the environment.

Greenspaces like parks, trails, gardens, and tree-covered areas have been associated with numerous health benefits, including improved mental health, increased physical activity, reduced stress, and lower all-cause mortality [8]. Epidemiologic studies have linked residential greenness to lower cardiovascular mortality, hypertension, and stroke [9]. Proposed mechanisms include increased physical activity, lower air and noise pollution, mitigation of urban heat, and restoration of psychological and physiological stress responses through parasympathetic activation [7,10,11]. Although the relationship between greenspace and coronary outcomes is documented, findings remain inconsistent across geographic contexts. Some studies report favorable associations, while others find weaker relationships, suggesting residual confounding or contextual modification [12,13], likely due to inconsistent measures of greenspace. Most existing research relies on static satellite-derived vegetation indices (e.g., Normalized Difference Vegetation Index, NDVI) as a proxy for “exposure” to nature. Such measures, while capturing the presence and coverage of greenery, leave out actual usage; a distinction increasingly recognized as critical to translate environmental exposures to tangible health benefits [14]. Recent evidence suggests that the cardioprotective potential of greenspace depends on patterns of use and behavior and may operate through cardiometabolic and behavioral pathways rather than direct physiological effects alone [10,[15], [16], [17]].

New mobility technologies now allow for the measurement of greenspace use through anonymized mobility data. Such metrics offer a behavioral complement to traditional metrics by capturing real-world engagement and can reveal variations in behavioral engagement across cities and neighborhoods. Integrating mobility-based measures with health surveillance data provides an opportunity to refine our understanding of how environmental engagement relates to chronic disease outcomes like CHD.

To address these gaps, the present study expands on prior research by focusing on behavioral greenspace engagement, rather than green coverage alone, and by evaluating how contexts shape their associations with CHD prevalence at the neighborhood level. We further examine how these associations differ across and within major U.S. metropolitan areas, accounting for variations in demographic composition, social and cultural vulnerabilities, and greenspace coverage (NDVI).

## 2 Methods

This ecological, cross-sectional study examined the association between greenspace visitation and CHD prevalence across the largest nine major U.S. metropolitan statistical areas (MSAs): Atlanta, Chicago, Dallas, Houston, Los Angeles, New York City, Philadelphia, Phoenix, and Washington, DC. MSAs were defined using the U.S. Census Bureau’s 2020 delineation of metropolitan and micropolitan areas. We did not include Miami, the 10th largest MSA, due to the absence of census tract-level health estimates in CDC PLACES. For each metro, county Federal Information Processing Standards (FIPS) codes were obtained from the Census Bureau, and all constituent census tracts were selected. The number of counties per metro ranged from two (Phoenix, Los Angeles) to twenty-two (New York City), yielding 13,152 census tracts across the nine study metros (Table 1). All data were de-identified and aggregated at the tract level; therefore, institutional review board (IRB) approval and informed consent were not required. All analyses were conducted in R (v4.3.1).

**Table 1:** Metropolitan coverage by census tracts.

MSA / Counties	CDC Places	Mobility	Demographics	SVI	NDVI	Coverage (%)
Atlanta (29)	948	948	882	948	948	93
Chicago (13)	2167	2167	2119	2167	2147	97.8
DC (23)	1264	1264	1209	1264	1254	95.6
Dallas (11)	1309	1309	1223	1309	1309	93.4
Houston (10)	1071	1071	1004	1071	1056	93.7
Los Angeles (2)	2904	2904	2893	2903	2892	99.6
NYC (22)	4138	4138	4097	4138	4138	99
Philadelphia (11)	1464	1464	1434	1464	1464	98
Phoenix (2)	984	984	936	984	984	95.1

Values represent the number of census tracts within each metropolitan statistical area with available data from each source. “Tracts with Complete Data (%)” indicates the proportion of census tracts with non-missing data across all datasets used in the analysis (CDC PLACES, greenspace visits from Advan Research, NDVI, SVI, and ACS demographics).

### 2.1 Data sources and variables of interest

Tract-level estimates of CHD and cardiometabolic risk factors were obtained from the CDC PLACES Project (2023). PLACES provides modeled small area estimates of chronic disease outcomes and risk factors derived from the Behavioral Risk Factor Surveillance System (BRFSS) and U.S. Census data. The outcome variable was CHD prevalence, while obesity (%), diabetes (%), and hypertension (%) were included as covariates along with social vulnerabilities (SVI) from CDC, demographics from ACS (2021), and NDVI. These cardiometabolic variables were included as neighborhood-level indicators of underlying health risk.

Greenspace engagement was quantified using 2021 mobility data from Advan Research (formerly SafeGraph), which aggregates anonymized smartphone location data across the United States. Greenspace points of interest (POIs) were defined using the U.S. Park Serve and OpenStreetMap land-use databases, restricted to public-access parks and recreational areas. To improve interpretability, Greenspace visits were calculated per person, and then scaled such that one unit represents ten visits per person:

$$\text{Greenspace}\;\text{visits}\;\text{per}\;\text{person}\;\left(\text{per}\;10\;\text{visits}\right)\;=\;\frac{\text{Total}\;\text{Greenspace}\;\text{Visits}}{\text{Number}\;\text{of}\;\text{Resident}\;\text{Devices}}\;/\;10$$

### 2.2 Environmental and social covariates

Environmental greenness was represented using the Normalized Difference Vegetation Index (NDVI, 2021), derived from Landsat 8 imagery [18]. Twelve monthly raster files (8th day of each month in 2021) were downloaded from NASA EarthData, stacked, and averaged to create a yearly composite. Mean NDVI values were extracted at the tract level using 2010 TIGER/Line shapefiles and zonal statistics. Values below zero (representing water or impervious surfaces) were set to zero. Neighborhood-level social and economic context was measured using the CDC/ATSDR Social Vulnerability Index (SVI, 2020 release). The index incorporates 15 Census variables grouped into four themes: socioeconomic status, household composition, minority status and language, and housing/transportation [19]. We included each of the four theme-specific indices with each theme score ranging from 0 to 1 (least to most vulnerable). The four SVI themes collectively incorporate multiple indicators related to socioeconomic status, education, employment, housing burden, disability, minority status, and transportation vulnerability. Demographic variables were obtained from the American Community Survey (ACS, 2021 5-year estimates), including percent female, percent non-Hispanic Black population, and median age. All datasets were harmonized to 2010 US census tract boundaries and merged using FIPS GEOID identifiers (Table 2).

**Table 2:** Data sources overview & variables of interest.

Dataset and Sources	Year of release	Variables Used
Greenspace visits (Advan Research/SafeGraph)	2021	Greenspace Visits, Number of Resident Devices
CHD and Metabolic Data (CDC Places)	2023	Coronary Heart Disease, Obesity, Hypertension, Diabetes
Social Vulnerabilities (CDC/ATSDR)	2020	Socioeconomic Status, Household Composition, Minority Status & Language, Housing & Transportation
Demographics (American Community Survey)	2021 5-Year Estimates	% Female, % Black, Median Age
Environmental Greenness (NDVI) (NASA EarthData)	2021	Annual Mean NDVI

CDC: Centers for Disease Control and Prevention (CDC), RWJF: Robert Wood Johnson Foundation (RWJF); ATSDR: Agency for Toxic Substances and Disease Registry; ACS: American Community Survey (conducted by the U.S. Census Bureau); NASA EarthData: NASA’s Earth Science Data Systems open access archive for earth science data.

### 2.3 Statistical analysis

All analyses were conducted at the census-tract level within each of the nine metropolitan statistical areas (MSAs), with MSAs defined by the Office of Management and Budget (OMB) [20]. After integrating variables from multiple data sources and excluding census tracts with missing data, our final analytic sample retained over 95 % of all tracts across the nine MSAs.

For each MSA, ordinary least squares (OLS) regression estimated the association between CHD prevalence and greenspace visits per person (per 10 visits), adjusting for demographics (percent female, percent non-Hispanic Black, median age), the four SVI 2020 themes, NDVI 2021, and metabolic factors (obesity, diabetes, and hypertension prevalence). Coefficients represent the change in CHD prevalence corresponding to a 10-visit-per-person increase in greenspace visit. In addition to metro-specific OLS models, a pooled regression framework was estimated to compare baseline CHD prevalence across metros. Model A provided an unpooled estimate of the overall association between greenspace visits and CHD, while Model B introduced fixed effects for metro areas to quantify between-city differences after adjusting for all covariates.

To evaluate spatial heterogeneity, we applied geographically weighted regression (GWR) to the fully adjusted model within each metro. GWR extends conventional regression by allowing associations between greenspace visitation and CHD prevalence to vary spatially rather than assuming a single metropolitan wide effect. The model fits a local regression at each census tract, weighting nearby tracts more heavily, which enables the estimation of location-specific coefficients that reflect neighborhood-level variation in the strength and direction of the association. Resulting outputs showed the local coefficients and R² values to identify variations in strength and direction of the associations.

In a secondary, exploratory analysis, we implemented a mediation analysis to assess whether neighborhood-level obesity, diabetes, and hypertension statistically accounted for variation in the association between greenspace visitation and CHD prevalence. This analysis used a regression-based mediation framework consistent with standard approaches for examining indirect statistical associations in observational data. Given the cross-sectional study design and the absence of temporal ordering between exposure, cardiometabolic factors, and outcome, these analyses were interpreted descriptively and were not intended to estimate or establish causal mediation. These analyses were conducted using sequential modeling approaches to examine whether inclusion of cardiometabolic variables altered the association between greenspace visitation and CHD. All analyses were performed in R Studio 2025.05. GWR models were estimated using R packages like spgwr and census tract–level spatial processing and visualization handled through sf, Tigris, and tmap.

## 3 Results

**Fig. 1:**
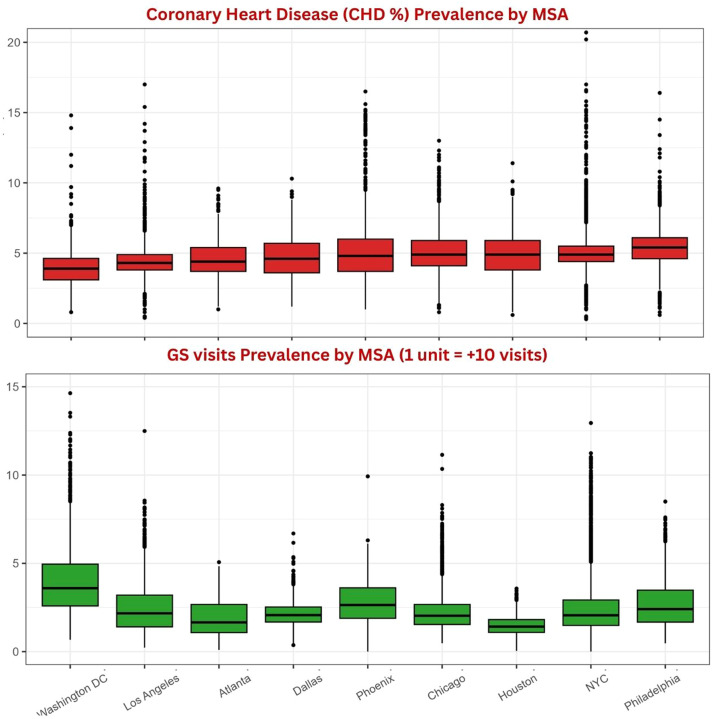
Distribution of Coronary Heart Disease Prevalence and Greenspace Visitation Across Nine U.S. Metropolitan Areas.Boxplots summarize census tract–level distributions, showing medians, interquartile ranges, and outliers. Coronary heart disease (CHD) prevalence is derived from CDC PLACES and represents the modeled % of adults who report ever being told by a health professional that they had angina or coronary heart disease. Greenspace visits per person are calculated using Advan Research mobility data normalized by resident device counts.

The analytic sample included 13,152 census tracts (96.13 % of total) across nine major U.S. metropolitan areas, representing approximately 72 million residents. CHD prevalence varied substantially across cities, with median (IQR) values ranging from 3.9 % (3.1–4.6 %) in Washington, DC to 5.4 % (4.6–6.1 %) in Philadelphia (Fig. 1). Greenspace visitation also differed across metros, with higher engagement in Phoenix, DC, and Atlanta and lower levels in New York City and Los Angeles. The considerable variation in both CHD prevalence and greenspace visitation across and within metros motivated the use of metro-specific regression models and spatial analyses to better capture patterns in local associations.

### 3.1 Linear regression

**Fig. 2:**
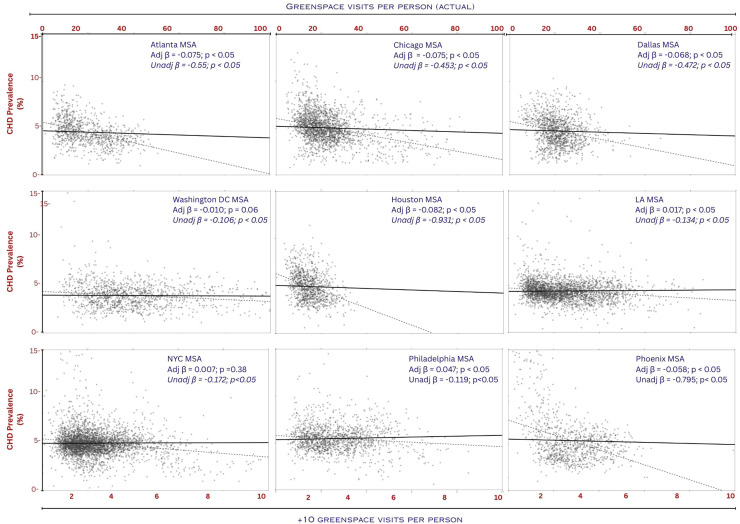
Results from adjusted and unadjusted linear associations between greenspace visitation and coronary heart disease prevalence across census tracts in nine major U.S. metropolitan are. Each point represents a census tract. Red lines indicate fitted ordinary least squares (OLS) regression lines adjusted for vegetation (NDVI), social vulnerability (SVI themes), and demographic covariates. Negative slopes indicate lower coronary heart disease prevalence with higher greenspace engagement.

Table 3 summarizes the fully adjusted linear regression models examining the association between 10 greenspace visits and CHD prevalence, adjusting for demographics, SVI, NDVI, and metabolic factors. Negative coefficients (Fig. 2) indicate a favorable association, meaning higher greenspace visitation corresponds with lower CHD prevalence, whereas positive coefficients indicate the opposite pattern. Across the nine metropolitan areas, greater greenspace visitation was associated with lower CHD prevalence in six cities. In Houston, each additional 10 visits per person was associated with a 1.65 % lower CHD prevalence (β = −0.082, *p* = 0.002). Similar patterns were observed in Atlanta (−1.64 %, β = −0.075, *p* < 0.001) and Dallas (−1.45 %, β = −0.068, *p* < 0.001), Chicago (−1.50 %, β = −0.075, *p* < 0.001) and Phoenix (−1.09 %, β = −0.058, *p* = 0.004). Associations were not significant in New York City (0.14 %, β = 0.007, *p* = 0.38) and Washington, DC (−0.25 %, β = −0.010, *p* = 0.06), whereas the opposite pattern emerged in Los Angeles (0.39 %, β = 0.017, *p* = 0.006; 0.39 %) and Philadelphia (0.86 %, β = +0.047, *p* < 0.001).

**Table 3:** Regression results: green space visits - coronary heart disease prevalence by metropolitan area.

Metro	β (per 10 visits/person)	95 % CI	p-value	Adj. R²
Atlanta	−0.075	(−0.1058, −0.0451)	<0.001	0.682
Chicago	−0.075	(−0.0934, −0.0606)	<0.001	0.707
Dallas	−0.068	(−0.0958, −0.0373)	<0.001	0.693
Houston	−0.082	(−0.1328, −0.0306)	0.002	0.713
Phoenix	−0.058	(−0.0960, −0.0200)	0.004	0.645
NYC	+0.007	(−0.0088, 0.0231)	0.381	0.661
LA	+0.017	(+0.0049, 0.0285)	0.006	0.649
Philadelphia	+0.047	(+0.0361, 0.0572)	<0.001	0.687
DC	−0.010	(−0.0206, 0.0003)	0.0629	0.642

Values shown represent the regression coefficient (β) for greenspace visits from multivariable ordinary least squares models. All models are adjusted for normalized difference vegetation index (NDVI), four Social Vulnerability Index (SVI) theme scores, and demographic covariates (percentage female, percentage Black, and median age). Confidence intervals and p-values correspond to the greenspace visits coefficient only.

To complement metro-specific models, we also conducted a pooled OLS analysis to estimate between-city differences in adjusted baseline CHD prevalence. In Model A (unpooled), each additional 10 greenspace visits per person were associated with a miniscule increase in CHD prevalence (β = 0.0091, 95 % CI = 0.0022 to 0.016, *p* = 0.010) (Supplementary Figure S1-S2). When metro-level fixed effects were added (Model B), the association modestly strengthened (β = 0.0133, 95 % CI = 0.0069 to 0.0197, *p* < 0.001), improving model fit and highlighting substantial between-city variation. Relative to Atlanta (reference), the largest differentials occurred in Phoenix (+0.99 pp), Philadelphia (+0.82 pp), Chicago (+0.73 pp), and New York City (+0.66 pp), each significantly higher (*p* < 0.001). Houston (+0.19 pp), Dallas (+0.17 pp), and Los Angeles (+0.09 pp) were moderately higher, while Washington DC (+0.03 pp) was statistically indistinguishable from Atlanta (*p* = 0.13). Overall, all metros except DC exhibited significantly higher adjusted baseline CHD burden than Atlanta, with the largest residual risks observed in Phoenix and Philadelphia (Supplementary Table S1).

The pooled models capture both within-city and between-city variation in CHD prevalence and greenspace visits and therefore primarily reflect differences across metropolitan contexts. As such, their direction should not be interpreted as representative of within-city associations. In contrast, the metro-specific models isolate within-city variation and more directly reflect how differences in greenspace visitation relate to CHD prevalence within individual metropolitan areas.

### 3.2 Spatial variations and patterns

While the linear models quantified overall metro-level associations by treating each system as spatially uniform, urban health dynamics are rarely homogenous. Therefore, we conducted GWR models to assess how the magnitude and direction of the greenspace–CHD association varied across tracts within each city.

**Fig. 3:**
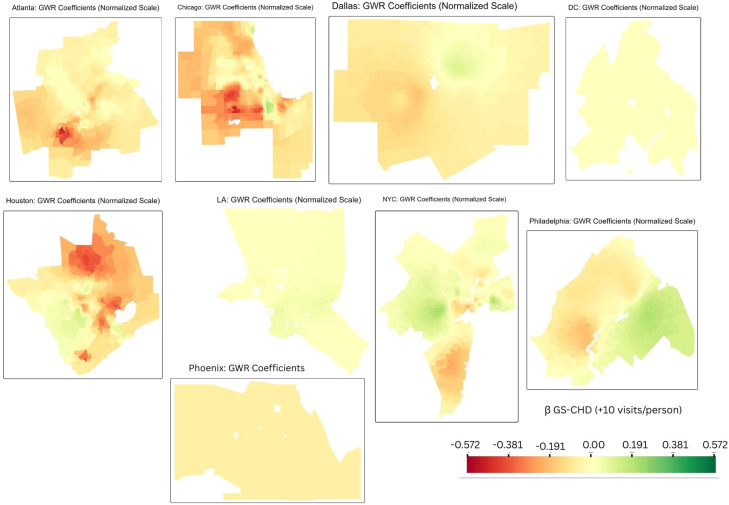
Spatial variation in the association between greenspace visitation and coronary heart disease (CHD) prevalence across census tracts in nine major U.S. metropolitan areas, estimated using geographically weighted regression (GWR). Values represent local regression coefficients (β). Maps display local GWR coefficients estimating the association between greenspace visits and coronary heart disease prevalence at the census tract level. Negative coefficients (red shades) indicate areas where higher greenspace engagement is associated with lower prevalence of CHD, whereas positive coefficients (green shades) indicate weaker or adverse associations.

**Fig. 4:**
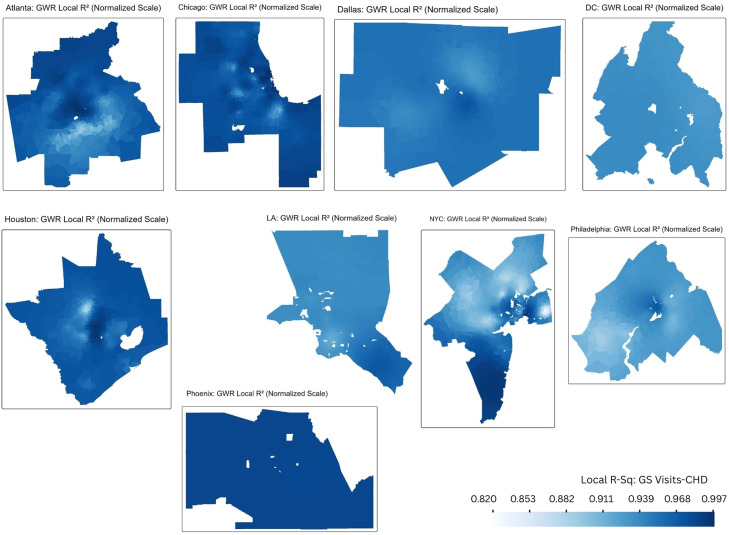
Spatial variation in model explanatory power (R²) from geographically weighted regression assessing the association between greenspace visitation and coronary heart disease prevalence. Local R² values represent the proportion of variance in CHD prevalence explained by the GWR model at each census tract. Darker shades indicate stronger local model performance, highlighting areas where greenspace engagement and covariates better capture neighborhood-level variation.

GWR revealed marked within-metro variation in both effect size and model fit, underscoring the localized nature of the greenspace-CHD relationship (Fig. 3, Fig. 4). Across metros, inverse association gradients were spatially concentrated and context dependent. The strongest association between higher green space visitation and lower CHD prevalence was observed in Houston, Atlanta, and Chicago, with strongest associations particularly in southern Harris County, southern Fulton/DeKalb Counties, and southwestern suburban Chicago (Kendall, Grundy, Will) displayed coefficients between −0.38 and −0.10. Dallas exhibited a weaker but favorable pattern, with coefficients mostly between −0.19 and 0.00. Slightly stronger associations emerged near Fort Worth and Arlington, while northern suburban tracts around Plano and Greenville showed neutral or mildly positive slopes. In New York, both the urban core (5 boroughs) and coastal New Jersey counties (Monmouth, Ocean) demonstrated beneficial gradients, whereas outer suburban tracts in Morris, Hunterdon, and Nassau Counties were weakly positive.

By contrast, Philadelphia displayed a mixed to unfavorable pattern, with southern New Jersey showing positive coefficients and only isolated pockets in central Philadelphia and northern New Castle County. Washington, DC, Los Angeles, and Phoenix were largely null, with coefficients near zero across nearly all tracts. DC and Phoenix were uniformly light yellow, while LA exhibited small positive clusters near Long Beach and Torrance.

Model fit was high overall (0.7–1.0), peaking in areas where coefficients were strongest like central Harris County in Houston, southern Fulton/DeKalb in Atlanta, and the New Jersey coastal corridor (Fig. 4). These findings indicate that while greenspace engagement generally aligns with lower CHD prevalence, its strength and direction vary sharply by geography.

Secondary analyses suggested that differences in neighborhood cardiometabolic risk help explain part of the observed association between greenspace visitation and CHD prevalence, while the primary adjusted associations remained similar in direction (Supplementary Table S2).

## 4 Discussion

This study provides one of the first multi-city, tract-level analyses linking greenspace engagement to CHD prevalence across major U.S. metropolitan areas. While prior research has largely relied on vegetation indices such as NDVI to characterize greenspace exposure, our findings show that actual usage patterns offer complementary behavioral insights. Consistent with previous ecological and cohort studies that have found associations between neighborhood greenness and lower cardiovascular mortality, hypertension, and ischemic heart disease [9,21,22], our results reinforce that higher greenspace visits were generally associated with lower CHD prevalence, though the magnitude and sometimes direction of these associations varied geographically [9,21,22].

City-level OLS models revealed inverse and favorable associations in most metros, particularly Houston, Atlanta, and Chicago. While the metro-specific models demonstrated inverse associations between greenspace visitation and CHD prevalence, the pooled model yielded a modest positive association. This divergence between the two sets of estimates highlights the importance of distinguishing between within-city and between-city effects in ecological analyses. In the pooled fixed-effects model, several metropolitan areas demonstrated substantially higher adjusted baseline CHD prevalence relative to Atlanta (reference), particularly Phoenix (+0.99 pp), Philadelphia (+0.82), Chicago (+0.73), and New York City (+0.66). These findings suggest that the pooled association is influenced by structural differences across metropolitan areas and therefore should not be interpreted as representative of within-city relationships.

Supplementary single mediation analyses indicated that adjustment for hypertension and diabetes attenuated the association between greenspace visitation and CHD prevalence, suggesting that cardiometabolic risk may account for part of the observed relationship. This aligns with evidence from prior mechanistic studies showing that nature exposure is linked to lowering stress, supporting physical activity, and regulating autonomic and inflammatory pathways that precede metabolic dysfunction [6,23].

Spatial analyses further highlighted that these associations are not uniform within cities. GWR revealed highly localized favorable gradients especially in areas with greater social vulnerability or cardiometabolic burden underscoring the context dependence of environmental health benefits. In metros like Houston, Atlanta, and Chicago, neighborhoods with both high CHD prevalence and greater greenspace use demonstrated the strongest inverse relationships, suggesting that access, engagement and other contextual factors such as park quality, walkability, and safety are jointly correlated to improving CHD outcomes.

The absence of strong inverse associations in some metropolitan areas, such as Los Angeles and Philadelphia, may reflect unmeasured contextual factors. Environmental conditions including extreme heat, limited greenspace accessibility, or differences in built-environment infrastructure may influence how residents engage with outdoor spaces and, in turn, shape observed associations. In addition, patterns of time-use substitution may play a role. In settings characterized by extreme heat or seasonal discomfort, individuals may preferentially engage in indoor or structured physical activities (e.g., gyms, recreational centers, or home-based exercise), which may also confer cardiometabolic benefits. Such behavioral shifts could reduce reliance on outdoor greenspace use, contributing to weaker or inconsistent associations between greenspace visitation and CHD prevalence at the neighborhood level. Because these factors were not directly measured in the present study, these explanations should be viewed as hypotheses for future research rather than definitive interpretations of our findings.

Moreover, cities characterized by warmer or more humid climate regimes (e.g., Houston, Atlanta, Phoenix) displayed narrower visitation ranges, whereas cities with stronger seasonal patterns (e.g., New York, Chicago, Los Angeles) showed a wider spread of greenspace engagement. These patterns may suggest that local environmental conditions including climate, greenspace availability, and seasonal comfort could influence how frequently residents use greenspaces, contributing in part to the heterogeneity observed across cities. These interpretations should be considered hypothesis-generating and warrant further investigation, as these factors were not directly measured in the present analysis.

Together, these findings suggest that behavioral engagement with greenspaces is associated with neighborhood CHD prevalence, with substantial heterogeneity across metropolitan and neighborhood contexts. The observed associations should be understood within the broader landscape of behavioral and cardiometabolic risk.

### 4.1 Strengths and limitations

A major strength of this study is its integration of multiple high-resolution datasets. Including Advan mobility traces, CDC PLACES health estimates, NDVI, SVI, and demographics across nine of the largest U.S. metropolitan areas, our study leverages a large sample (∼13,000 tracts; ∼72 million residents) and multi-scalar design allow for both metro-level inference and neighborhood-scale spatial exploration. In particular, the use of mobility-based greenspace visitation represents a novel behavioral metric, addressing a key limitation of prior studies that rely solely on static vegetation measures and do not capture actual engagement with greenspaces.

Our study has a few limitations that merit consideration. First, the ecological and cross-sectional design precludes causal inference and may mask individual-level behavioral heterogeneity. Second, CHD estimates are modeled at the tract level and may not capture diagnostic or reporting variability. Third, while the mobility data provide relative visitation intensity, they cannot distinguish activity or greenspace type, duration, or purpose. Additionally, some areas with meaningful local greenspace such as large private yards, vegetated buffers, informal trails, or small community green areas may not be designated as POIs or classified as greenspace in our data sources, potentially underestimating true neighborhood access or usage. Fourth, potential unmeasured confounders including healthcare access, aspects of urban form, pollution, diet, and other unmeasured outdoor or indoor activities may contribute to residual bias.

Because this study is cross-sectional and based on prevalent CHD, reverse causation and health selection cannot be ruled out. Individuals with better underlying health or greater functional capacity may be more likely to engage in greenspace visitation, which could contribute to the observed inverse associations. Accordingly, these findings should be interpreted as associational patterns rather than evidence of a causal effect.

## 5 Future scope

Future research should extend these findings by integrating longitudinal and individual-level designs to clarify the causal pathways linking greenspace engagement to cardiovascular health. Prospective cohorts or wearable-sensor studies could disentangle whether increases in park use preceded improvements in blood pressure, glycemic control, or lipid profiles, or whether healthier individuals are more likely to engage with nature. Combining mobility data with clinical outcomes (e.g., EHR-based CHD incidence, biomarker data, or medication use) would strengthen causal inference and reveal dose-response relationships. At the population level, integrating greenspace quality, accessibility, and safety metrics with behavioral visitation patterns could help explain the spatial heterogeneity observed across cities. Future studies should also consider seasonal climate effects such as heat exposure in arid metros like Phoenix and Los Angeles and their influence on outdoor behavior and cardiovascular stress.

Finally, translating these insights into practice requires aligning urban design, public health, and health preventive strategies. Interventions that promote equitable greenspace use through active transportation networks, park-based physical activity programs, and targeted outreach to high-risk communities could help position greenspace as an active component of cardiovascular prevention strategies.

## Data availability statement

The datasets used in this study were obtained under a paid license from Advan Research (formerly SafeGraph) and are not publicly available. CDC PLACES, Social Vulnerability Index, ACS demographics, and NDVI data are publicly available from their respective repositories. Access to mobility data requires a commercial agreement with Advan Research.

## Disclosures

None.

## Statement of funding

No funding was acquired for this study.

## CRediT authorship contribution statement

**Sarayu Chandra Mouli:** Writing – review & editing, Writing – original draft, Validation, Project administration, Methodology, Investigation, Formal analysis, Data curation, Conceptualization. **Tong Zhang:** Writing – review & editing, Writing – original draft, Methodology. **Jad Ardakani:** Writing – review & editing, Writing – original draft, Methodology. **Zhuo Chen:** Writing – review & editing, Writing – original draft, Methodology. **Sanjay Rajagoaplan:** Writing – review & editing, Writing – original draft, Methodology. **Jay Maddock:** Writing – review & editing, Writing – original draft, Methodology. **Khurram Nasir:** Writing – review & editing, Writing – original draft, Validation, Methodology. **Weichuan Dong:** Writing – review & editing, Writing – original draft, Validation, Supervision, Resources, Project administration, Methodology, Investigation, Formal analysis, Data curation, Conceptualization. **Sadeer Al-Kindi:** Writing – review & editing, Writing – original draft, Validation, Supervision, Resources, Project administration, Methodology, Investigation, Conceptualization.

## Declaration of competing interest

The authors declare that they have no known competing financial interests or personal relationships that could have appeared to influence the work reported in this paper.

## Appendix Supplementary materials

Image, application 1

Image, application 1

Image, application 2

Image, application 3
