# Supplementary figures and images for: Neighborhood green space visits and coronary heart disease: Evidence from mobility data across nine U.S. metropolitan cities
Source: Am J Prev Cardiol. 2026 May 14;28:101666. doi: 10.1016/j.ajpc.2026.101666 (PMC13326116; doi:10.1016/j.ajpc.2026.101666)

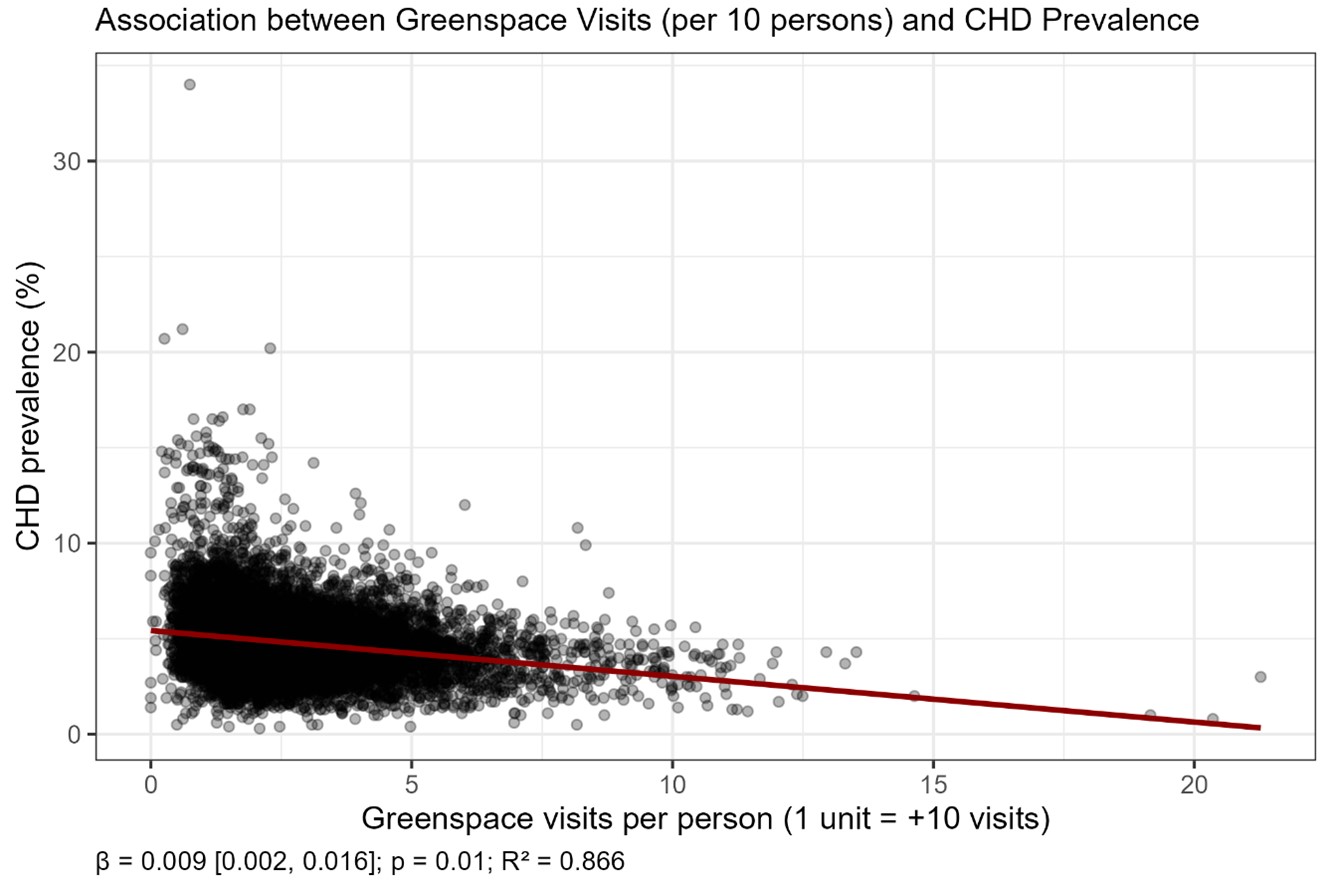

Supplement: Supplementary file 1 [file mmc1.zip › Supplementary fig1.jpg]

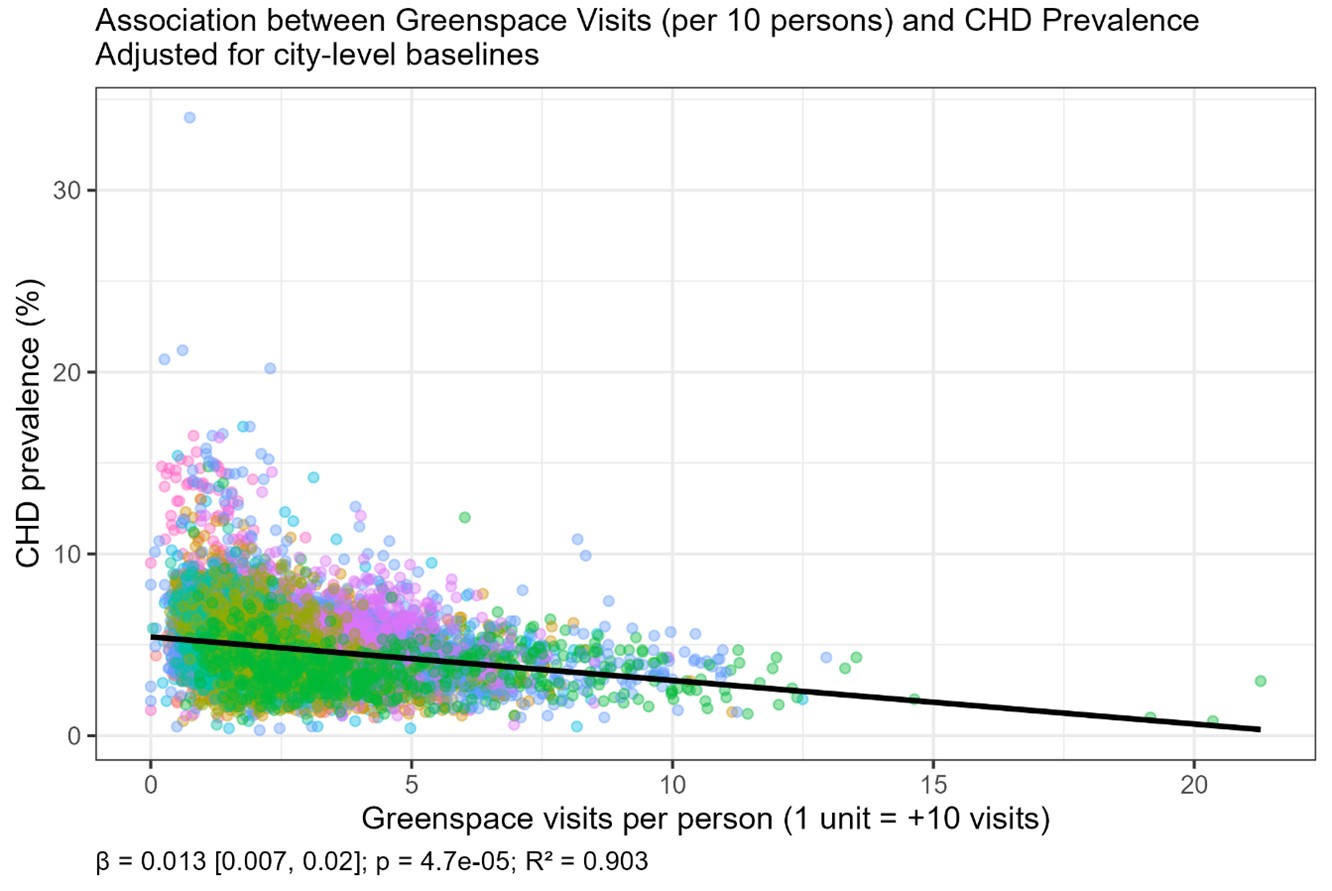

Supplement: Supplementary file 4 [file mmc4.zip › Supplementary fig2.jpg]
