# Supplementary material for: Neighborhood green space visits and coronary heart disease: Evidence from mobility data across nine U.S. metropolitan cities
Source: Am J Prev Cardiol. 2026 May 14;28:101666. doi: 10.1016/j.ajpc.2026.101666 (PMC13326116; doi:10.1016/j.ajpc.2026.101666)
Supplement: Supplementary file 2 [file mmc2.docx]

**Supplementary Table S1: Overall Pooled OLS summary, with and without MSA**

| Model | Specification | Est (β) | 95% CI | p-value | Adj. R² |
| --- | --- | --- | --- | --- | --- |
| Model A | Pooled OLS without Metro Fixed Effects | +0.0091 | (0.002, 0.016) | 0.010 | 0.53 |
| Model B | Pooled OLS with Metro Fixed Effects | +0.0133 | (0.006, 0.019) | < 0.001 | 0.62 |
| Metro-Level CHD Differences | | | | | |
| Metro | | Est (**β)** | **95% CI** | **p-value** | |
| All MSA Combined | | +0.0091 | (0.0022, 0.0160) | 0.01 | |
| Atlanta | | - (reference) | **--** | -- | |
| Chicago | | 0.73 | (0.69, 0.78) | <0.001 | |
| Dallas | | 0.17 | (0.13, 0.21) | <0.001 | |
| DC | | 0.03 | (-0.01, 0.08) | 0.13 | |
| Houston | | 0.19 | (0.14, 0.23) | <0.001 | |
| Los Angeles | | 0.09 | (0.05, 0.13) | <0.001 | |
| New York City | | 0.66 | (0.62, 0.70) | <0.001 | |
| Philadelphia | | 0.82 | (0.78, 0.87) | <0.001 | |
| Phoenix | | 0.99 | (0.94, 1.04) | <0.001 | |

**Supplementary Table S2: Direct and Indirect pathways: Single Mediation Analysis**

| Metro | Mediator | Model Pathways | | | | P |
| --- | --- | --- | --- | --- | --- | --- |
|  |  | **Direct Effect** | **IDE** | **Tot**. **Effect** | **% Mediated** *(95% CI)* |  |
| Atlanta | High BP | -0.08 | -0.42 | -0.50 | 84 (-0.53, -0.31) | <0.001 |
|  | Diabetes | -0.07 | -0.42 | -0.49 | 85 (-0.51, -0.32) | <0.001 |
|  | Obesity | -0.15 | -0.35 | -0.50 | 71 (-0.46, -0.25) | <0.001 |
| Chicago | High BP | -0.04 | -0.21 | -0.25 | 83 (-0.30, -0.13) | <0.001 |
|  | Diabetes | -0.08 | -0.17 | -0.25 | 68 (-0.26, -0.09) | <0.001 |
|  | Obesity | -0.12 | -0.13 | -0.25 | 52 (-0.22, -0.04) | <0.01 |
| Dallas | High BP | -0.06 | -0.39 | -0.45 | 87 (-0.48, -0.31) | <0.001 |
|  | Diabetes | -0.15 | -0.30 | -0.45 | 67 (-0.39, -0.21) | <0.001 |
|  | Obesity | -0.16 | -0.29 | -0.45 | 64 (-0.35, -0.22) | <0.001 |
| DC | High BP | 0.03 | -0.10 | -0.07 | 149 (-0.17, -0.03) | <0.01 |
|  | Diabetes | 0.02 | -0.09 | -0.07 | 130 (-0.14, -0.04) | <0.01 |
|  | Obesity | -0.03 | -0.04 | -0.07 | 57 (-0.09, -0.02) | <0.05 |
| Houston | High BP | -0.06 | -0.79 | -0.85 | 93 (-0.90, -0.68) | <0.001 |
|  | Diabetes | -0.26 | -0.59 | -0.85 | 69 (-0.64, -0.53) | <0.001 |
|  | Obesity | -0.36 | -0.49 | -0.85 | 58 (-0.55, -0.43) | <0.001 |
| Los Angeles | High BP | -0.01 | -0.15 | -0.16 | 94 (-0.20, -0.09) | <0.001 |
|  | Diabetes | 0.18 | -0.34 | -0.16 | 213 (-0.42, -0.26) | <0.001 |
|  | Obesity | -0.09 | -0.07 | -0.16 | 46 (-0.11, -0.03) | <0.05 |
| New York City | High BP | 0.03 | -0.22 | -0.19 | 114 (-0.29, -0.15) | <0.001 |
|  | Diabetes | -0.06 | -0.13 | -0.19 | 67 (-0.18, -0.09) | <0.001 |
|  | Obesity | -0.11 | -0.08 | -0.19 | 41 (-0.12, -0.05) | <0.01 |
| Philadelphia | High BP | 0.11 | -0.33 | -0.22 | 152 (-0.39, -0.27) | <0.001 |
|  | Diabetes | -0.02 | -0.20 | -0.22 | 92 (-0.26, -0.15) | <0.001 |
|  | Obesity | -0.05 | -0.16 | -0.22 | 75 (-0.21, -0.12) | <0.001 |
| Phoenix | High BP | -0.05 | -0.49 | -0.54 | 91 (-0.56, -0.41) | <0.001 |
|  | Diabetes | -0.18 | -0.35 | -0.54 | 66 (-0.42, -0.28) | <0.001 |
|  | Obesity | -0.44 | -0.10 | -0.54 | 18 (-0.18, -0.03) | <0.05 |
