# Supplementary material for: Neighborhood green space visits and coronary heart disease: Evidence from mobility data across nine U.S. metropolitan cities
Source: Am J Prev Cardiol. 2026 May 14;28:101666. doi: 10.1016/j.ajpc.2026.101666 (PMC13326116; doi:10.1016/j.ajpc.2026.101666)
Supplement: Supplementary file 3 [file mmc3.docx]

**Supplementary Figure S1: Pooled Association Between Greenspace Visits and CHD Prevalence Without Metropolitan Fixed Effects**

Adjusted linear regression model examining the association between greenspace visits per person (per 10 visits) and coronary heart disease (CHD) prevalence across nine U.S. metropolitan statistical areas (MSAs), without inclusion of metro fixed effects. Models adjust for demographic characteristics, Social Vulnerability Index (SVI) themes, NDVI (2021), and cardiometabolic risk factors (diabetes, hypertension, and obesity). The figure displays the overall pooled slope and confidence interval.

**Supplementary Figure S2: Pooled Association Between Greenspace Visits and CHD Prevalence with Metropolitan Fixed Effects**

Adjusted pooled linear regression model including metropolitan fixed effects to account for between-city differences. The association between greenspace visits per person (per 10 visits) and CHD prevalence is estimated while adjusting for demographics, SVI themes, NDVI, and cardiometabolic risk factors. Inclusion of metro fixed effects isolates within metro associations.

**Supplementary Table S1: Pooled Ordinary Least Squares (OLS) Regression Models Across Nine MSAs**

Summary of pooled OLS regression models evaluating the association between greenspace visits per person (per 10 visits) and CHD prevalence across nine MSAs. Results are presented for models without metropolitan fixed effects and with metropolitan fixed effects. Models adjust for demographic covariates, SVI themes, NDVI, and cardiometabolic risk factors. Estimates include regression coefficients (β), 95% confidence intervals, p-values, and model fit statistics.

**Supplementary Table S2: Direct and Indirect Associations Between Greenspace Visits and CHD: Single Mediation Analysis**

Results from single mediation analyses examining whether the association between greenspace visits per person (per 10 visits) and CHD prevalence operates indirectly through cardiometabolic conditions, including hypertension, diabetes, and obesity. Total, direct, and indirect effects are reported with corresponding confidence intervals and p-values.
